# Suicidal Attempts in Psychiatric Patients admitted to “Xhavit Gjata” Hospital, Albania: A 2-year Retrospective Descriptive Study

**DOI:** 10.1192/j.eurpsy.2025.2352

**Published:** 2025-08-26

**Authors:** L. Demiraj, E. Sotiri, F. Elezi, E. Petrela

**Affiliations:** 1 Psychiatry; 2 Public Health (Biostatistic sector), UHC “Mother Teresa”, Tirana, Albania

## Abstract

**Introduction:**

Suicide represents one of the most discussed mental health issues in the world today and health challenges for the future. The burden of suicide is calculated in very high numbers (720.000 people per year) ranking it among the most frequent causes of death (World Health Organization. Suicide. WHO Fact Sheet. 2024 https://www.who.int/news-room/fact-sheets/detail/suicide). In the context of patients hospitalized in psychiatric services, the incidence of suicide attempts is particularly high, representing a major challenge for mental health professionals and the health care system.

**Objectives:**

This study aims to analyze the socio-demographic and clinical factors influencing suicide attempts in a sample of psychiatric patients in Albania and looks for statistically significant relationships between them.

**Methods:**

A retrospective study was conducted on 138 psychiatric patients admitted after a suicide attempt and data from August 2022 to July 2024 were obtained. Socio-demographic and clinical data were collected and analyzed. The relationship between these variables were explored. A total of 28 different demographic, clinical and behavioral variables were sampled and pooled with the help of statistical software.

**Results:**

From the data it was found that suicide attempts were more frequent among women with a woman/man ratio of 1.42:1, age 25-44 years and among unemployed persons during the working age. It was more frequent in urban areas, with an uban / rural ratio of 2.85:1. The education level most frequent was primary (8-years of education) in 44.2% of the cases. Our data showed that 86.2% of cases did not live alone, which can be explained by the traditional Albanian family structure. However, only 28.3% of cases had good family support. Suicide attempts were most common in summer. The most frequent discharge diagnosis was a mood disorder in 69.6% of the cases, while a co-diagnosis was present in only 22.5% of cases. 59.1% were hospitalized for a first attempt. The attempt was reported as premeditated in 64.5% of cases, with prior preparation in 21.7% of cases and without asking for help in 62.3% of cases. There is a significant relationship (p<0.05) between the diagnosis and seeking help. A significant relationship was found between compliance and first or repeated attempts and also between diagnosis and first or repeated attempts. The most frequently used type of attempted suicidal method was the use of medications in 41.3% of cases.

**Conclusions:**

The pattern presented in the study group in relation to the characteristics of patients who commit suicide attempts, is close to the patterns presented by similar studies. Differences and non-correlations are atributed to local factors. Identification of suicide behaviors pave the way for treatment and assistance for anyone considering suicide. Further research is needed to examine outpatient and community samples.

**Disclosure of Interest:**

None Declared

